# Supplementary material for: Species Identity Dominates over Environment in Driving Bacterial Community Assembly in Wild Invasive Leaf Miners
Source: Microbiol Spectr. 2022 Mar 28;10(2):e00266-22. doi: 10.1128/spectrum.00266-22 (PMC9045101; doi:10.1128/spectrum.00266-22)
Supplement: SUPPLEMENTAL FILE 1 — Supplemental material. Download SPECTRUM00266-22_Supp_1_seq12.pdf, PDF file, 1.0 MB [file spectrum00266-22_supp_1_seq12.pdf]

Supplemental Information for:

Species Identity Dominates over Environment in Driving Bacterial Community Assembly in Wild Invasive Leaf Miners

Yu-Xi Zhu[a], Ya-Wen Chang[a], Tao Wen[b], Run Yang[a], Yu-Cheng Wang[a], Xin-Yu Wang[a], Ming-Xing Lu[a], Yu-Zhou Du[a*]

[a]Institute of Applied Entomology, School of Horticulture and Plant Protection, Yangzhou University, Yangzhou, Jiangsu 225009, China.

[b]The Key Laboratory of Plant Immunity, Jiangsu Provincial Key Lab for Organic Solid Waste Utilization, Jiangsu Collaborative Innovation Center for Solid Organic Wastes, Educational Ministry Engineering Center of Resource-saving fertilizers, Nanjing Agricultural University, Nanjing 210095, China

***Correspondence** to Yu-Zhou Du, E-mail: yzdu@yzu.edu.cn; Address: Institute of Applied Entomology, School of Horticulture and Plant Protection, Yangzhou University, Yangzhou, Jiangsu 225009, China.

RUNNING TITLE: Bacterial community assembly in *Liriomyza*

This file includes:

Table S2 to S4；

Fig. S1 to S8.

Table S2 The alpha diversity of four leaf miner bacterial communities.

Host species	ACE	Chao 1	Pielou evenness	Simpson evenness	Shannon	Richness
L. chinensis	569.71 ± 300.50	526.96 ± 287.33	0.57 ±0.18	0.034 ±0.022	3.27 ±1.36	316.42 ± 190.04
L. huidobrensis	426.95 ± 289.73	406.77 ± 272.75	0.38 ±0.22	0.026 ±0.016	2.02 ±1.37	181.56 ± 126.59
L. trifolii	399.31 ± 288.21	365.67 ± 235.58	0.46 ±0.13	0.029 ±0.016	2.39 ±0.87	185.07 ± 101.59
L. sativae	428.23 ± 319.59	385.22 ± 271.22	0.46 ±0.14	0.035 ±0.038	2.41 ±1.01	193.90 ± 129.73

Table S3 Network features of bacterial community of laboratory and wild leaf miners.

Topological property	Laboratory	Wild
Edges	425	257
Positive edges	344	257
Negative edges	81	0
Vertices	67	65
Connectance	0.192221	0.123558
Average degree	12.68657	7.907692
Average path length	2.435097	1.884861
Diameter	4.13294	3.859676
Edge connectivity	1	0
Clustering coefficient	0.716957	0.934223
Clusters	1	6
Centralization degree	0.247173	0.079567
Centralization betweenness	0.136181	0.042124
Centralization closeness	0.270383	0.007344

Table S4 Redundancy analysis (RDA) variation.

Factors	Explained variation (%)	*F*	*p*
Sex	0.23	1.05	0.387
Host plant	1.10	2.61	0.001
Location	1.00	2.94	0.001
Longitude	2.33	12.70	0.001
AMT	1.90	7.83	0.001
AP	1.95	4.98	0.001
Species	1.17	3.90	0.001

AP: annual mean precipitation; AMT: annual mean temperature.

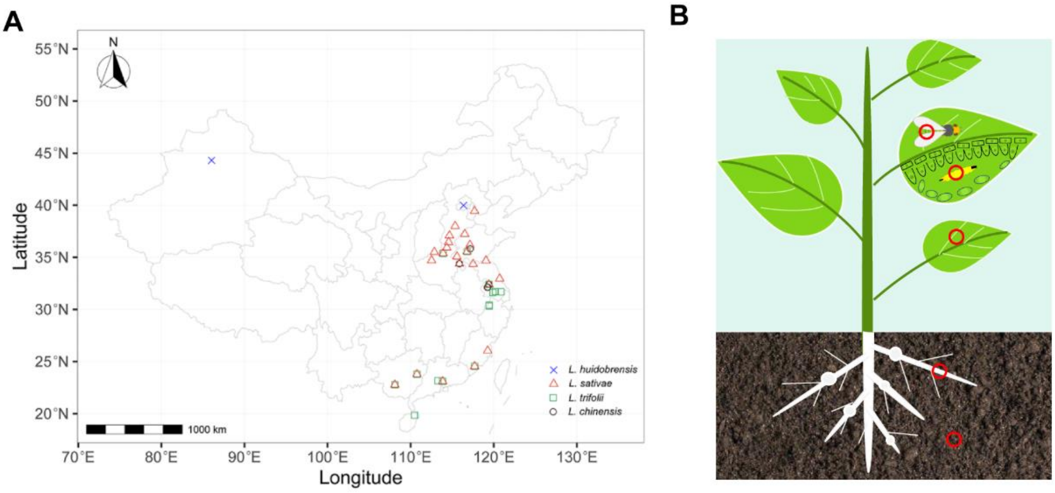

Leaf miner species	Populations	Host plants	Individuals
L. huidobrensis	2 (1 lab, 2 wild)	1	18 (9♀, 9♂)
L. sativae	23 (1 lab, 23 wild)	3	160 (79♀, 81♂)
L. trifolii	14 (1 lab, 14 wild)	5	113 (60♀, 53♂)
L. chinensis	4 (1 lab, 4 wild)	1	19 (11♀, 8♂)
Total	43	6	310

Host species	No. of samples
Adult (*L. sativae*)	8
Larva (*L. sativae*)	9
Leaf	8
Root	8
Soil	8

FIG S1 Experimental design. (A) Location of sampling sites across multiple geographic regions of China. A total of 310 individual samples associated with four leaf miner species, including *L. trifolii*, *L. sativae*, *L. chinensis* and *L. huidobrensis*, were collected from 43 populations and 6 host plant species. (B) Samples from the continuum of leaf miners and their associated with leaves, roots and soil. Detailed samples information corresponded in Table S1 in the Supplemental Material.

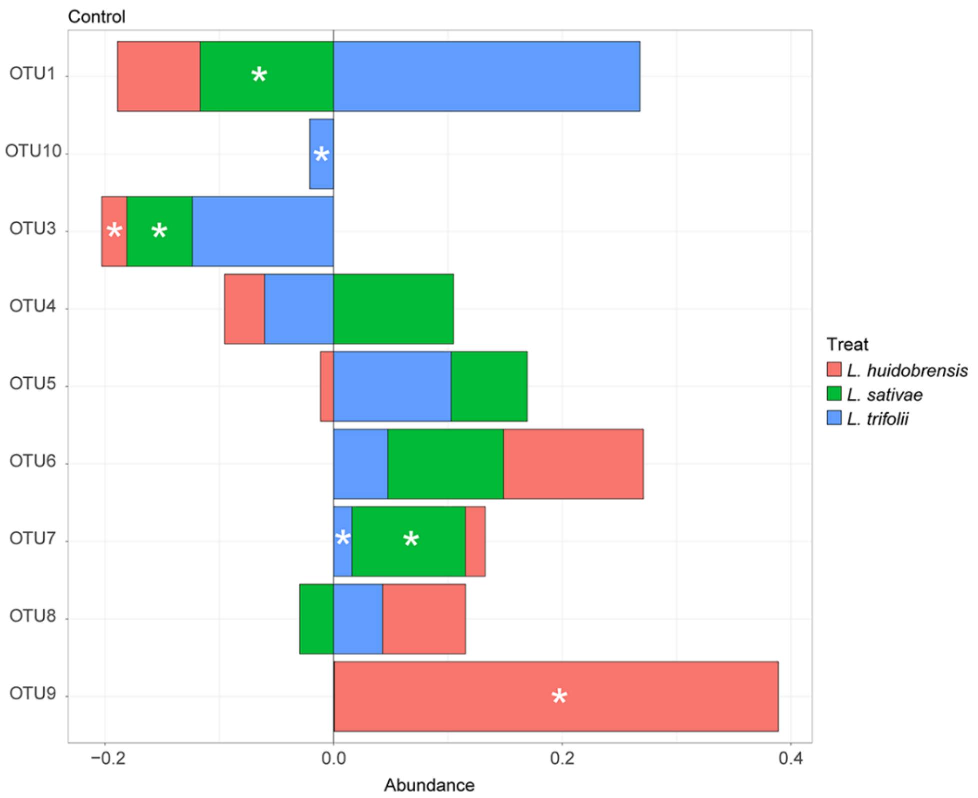

FIG S2 The significantly different OTUs between the native leaf miner *L. chinensis* and other three invasive leaf miners. "*" represents significant difference between each invasive leaf miner and the native leaf miner (Control) ($p < 0.05$).

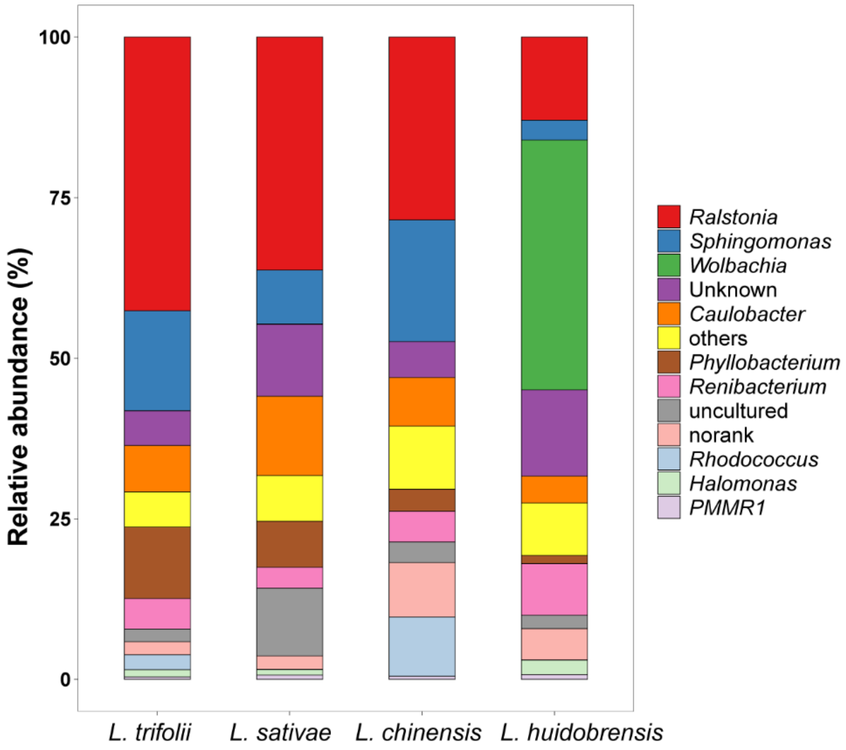

FIG S3 Relative abundance of each bacterial genus in different leaf miner species.

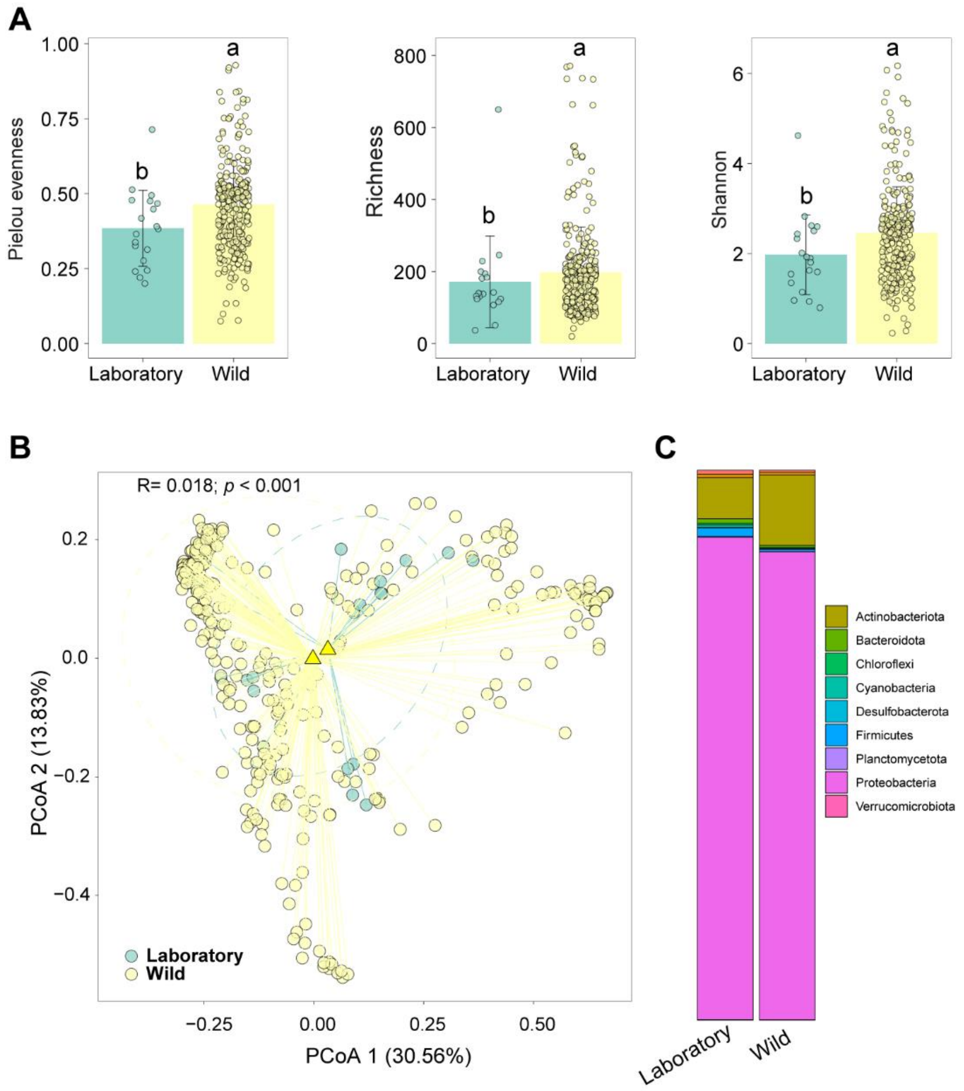

FIG S4 Comparison between the bacterial communities in laboratory-reared and wild leaf miner. (A) The alpha diversity indices (Pielou evenness, Shannon and Richness index) of the laboratory-reared and wild leaf miner bacterial communities. Different letters indicate significant differences between groups at a level of $p < 0.05$. (B) Principal coordinates analysis (PCoA) of bacterial community structure between the laboratory-reared and wild leaf miner. (C) The relative abundance of each bacterial phylum in the laboratory-reared and wild leaf miner.

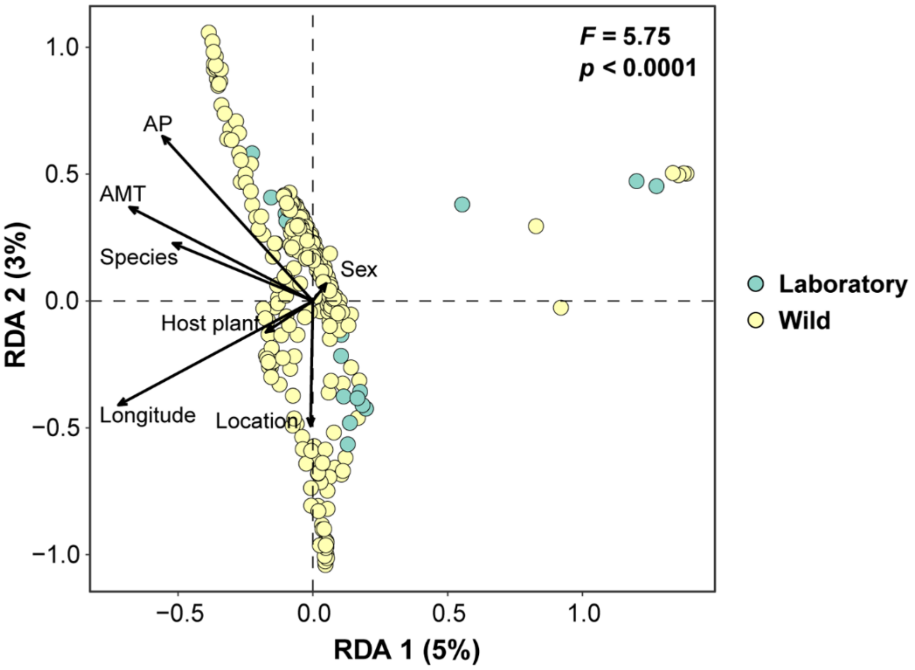

FIG S5 Redundancy analysis (RDA) highlighting correlations between multiple environmental factors and bacterial communities. AP: annual mean precipitation; AMT: annual mean temperature.

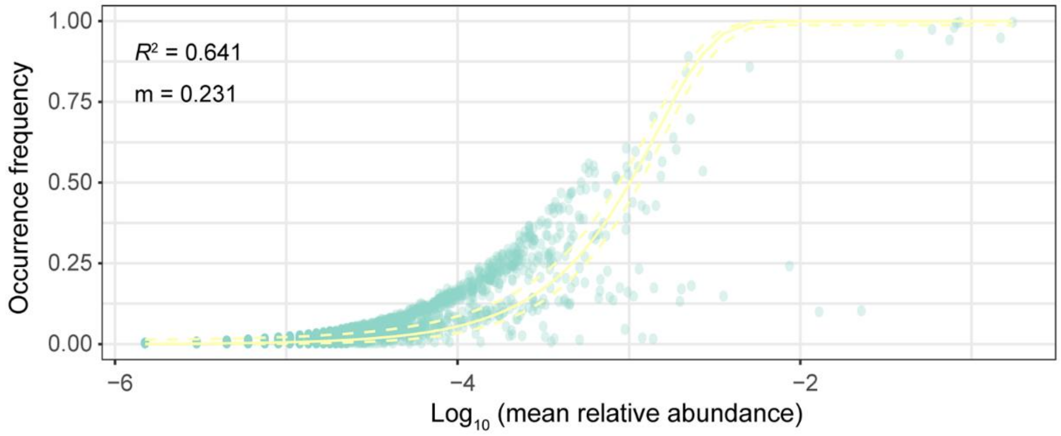

FIG S6 Fit of the neutral for 310 leaf miner samples. The yellow solid and dashed lines represent the predicted occurrence frequency and 95% confidence interval of the neutral model, respectively. *m* indicates the estimated migration rate and R^2 indicates the fit to the neutral model.

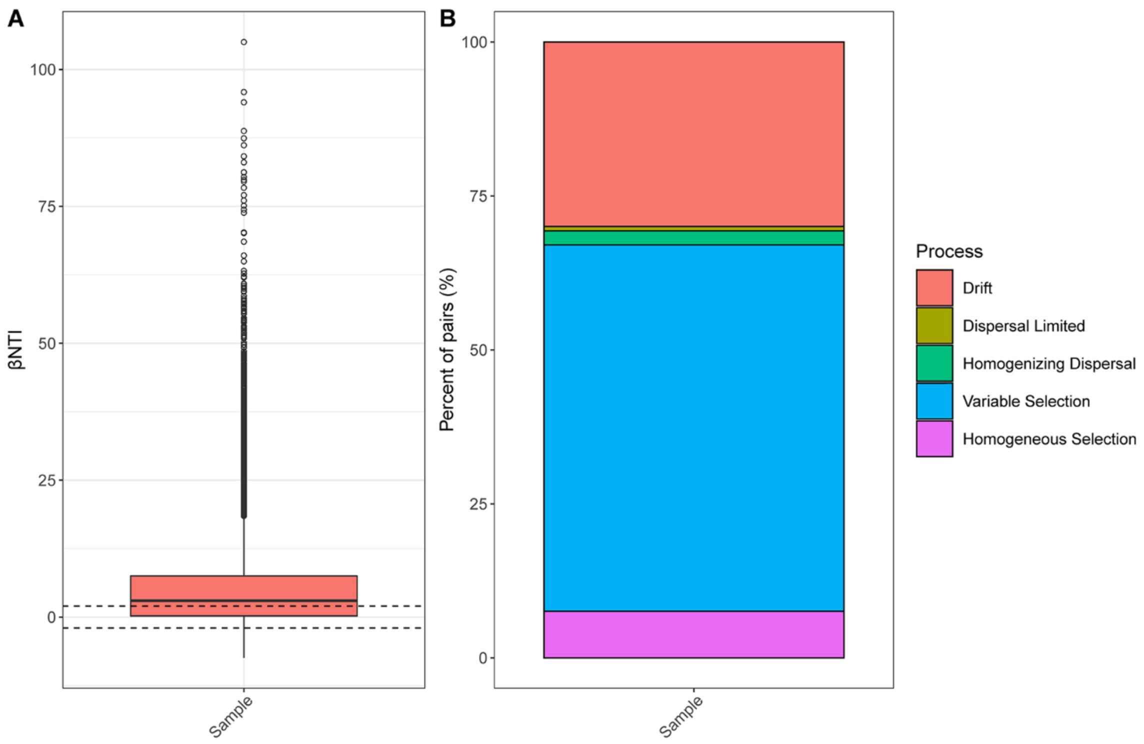

FIG S7 Ecological processes driving the assembly of bacterial communities in all samples. (A) Effects of deterministic (βNTI ≥ 2 or βNTI ≤ -2) and stochastic processes (-2 < βNTI < 2) in shaping bacterial community in all samples. (B) The quantified major ecological processes in governing the microbial community.

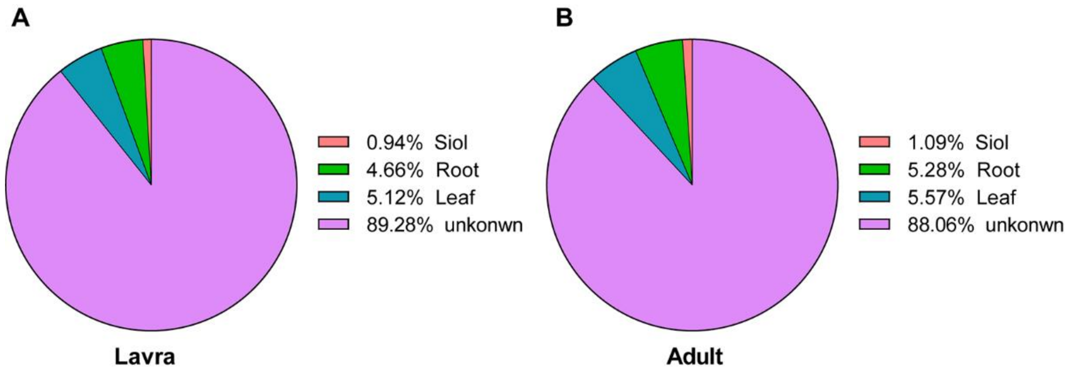

FIG S8 Source-tracking analysis of larva (A) and adult (B) of leaf miner microbes derived from leaf, root and soil.
